# Epigenetic silencing of EYA2 in pancreatic adenocarcinomas promotes tumor growth

**DOI:** 10.18632/oncotarget.1842

**Published:** 2014-03-22

**Authors:** Audrey Vincent, Seung-Mo Hong, Chaoxin Hu, Noriyuki Omura, Angela Young, Haeryoung Kim, Jun Yu, Spencer Knight, Michael Ayars, Margaret Griffith, Isabelle Van Seuningen, Anirban Maitra, Michael Goggins

**Affiliations:** ^1^ Department of Pathology, the Sol Goldman Pancreatic Cancer Research Center, Johns Hopkins Medical Institutions, Johns Hopkins University, Baltimore, MD, USA; ^2^ Department of Oncology, the Sol Goldman Pancreatic Cancer Research Center, Johns Hopkins Medical Institutions, Johns Hopkins University, Baltimore, MD, USA; ^3^ Department of Medicine, the Sol Goldman Pancreatic Cancer Research Center, Johns Hopkins Medical Institutions, Johns Hopkins University, Baltimore, MD, USA; ^4^ Inserm, UMR837, Jean-Pierre Aubert Research Center, Lille Cedex, France; ^5^ Université Lille Nord de France, Lille Cedex, France; ^6^ Centre Hospitalier Régional et Universitaire de Lille, Lille Cedex, France

**Keywords:** EYA2, pancreatic cancer, epigenetic

## Abstract

To identify potentially important genes dysregulated in pancreatic cancer, we analyzed genome-wide transcriptional analysis of pancreatic cancers and normal pancreatic duct samples and identified the transcriptional coactivator, EYA2 (Drosophila Eyes Absent Homologue-2) as silenced in the majority of pancreatic cancers. We investigated the role of epigenetic mechanisms of EYA2 gene silencing in pancreatic cancers, performed *in vitro* and *in vivo* proliferation and migration assays to assess the effect of EYA2 silencing on tumor cell growth and metastasis formation, and expression analysis to identify genes transcriptionally regulated by EYA2. We found loss of tumoral Eya2 expression in 63% of pancreatic cancers (120/189 cases). Silencing of EYA2 expression in pancreatic cancer cell lines correlated with promoter methylation and histone deacetylation and was reversible with DNA methyltransferase and HDAC inhibitors. EYA2 knockdown in pancreatic cancer cell lines increased cell proliferation. Compared to parental pancreatic cancer cells, pancreatic cancers stably-expressing EYA2 grew more slowly and had fewer metastases in orthotopic models. The transcriptional changes after stable expression of EYA2 in pancreatic cancer cells included induction of genes in the TGFbeta pathway. Epigenetic silencing of EYA2 is a common event in pancreatic cancers and stable expression EYA2 limits the growth and metastases of pancreatic adenocarcinoma.

## INTRODUCTION

Pancreatic cancer is the fourth leading cause of cancer-related death in the United States, with a dismal 5-year survival rate of ~ 5%. The majority of patients are diagnosed with advanced-stage disease, contributing to their poor prognosis [1]. There is a need to understand the molecular mechanisms responsible for pancreatic cancer development and progression in order to identify effective therapeutic targets for this disease and whenever possible ensure its early detection. Pancreatic ductal adenocarcinomas develop as a result of clonally-selected genetic events most commonly involving the genes *KRAS, CDKN2A, TP53* and *SMAD4* [2-6], less commonly *ATM* and others [3, 5, 7, 8], and for pancreatic ductal adenocarcinomas arising from intraductal papillary mucinous neoplasms, most commonly *KRAS, CDKN2A, TP53*, as well as *GNAS* and *RNF43* [9-11]. Previous studies have demonstrated that aberrant expression of epigenetically regulated genes contributes to pancreatic cancer development and progression [12-16]. To further identify epigenetically deregulated genes in pancreatic cancers, we compared the published SAGE (Serial analysis of gene expression) profiles of pancreatic ductal adenocarcinomas and normal pancreatic duct cells [3], focusing on silenced genes implicated in cancer progression that had not been reported as silenced in pancreatic cancer. From this analysis we identified Drosophila Eyes Absent Homologue 2 (*EYA2*) as a commonly silenced gene in pancreatic ductal adenocarcinomas.

Eya2 is normally expressed early during development and is characterized by two independent functions [17]. As a transcriptional coactivator, Eya2 is involved in the regulation of the retinal determination gene network, essential for eye fate specification, which plays an important role in regulating cell death and/or differentiation during development [18]. Additionally, the tyrosine phosphatase activity of Eya2 [18] has been shown to dephosphorylate H2AX, promoting repair and cell survival in the response to DNA damage [19]. Other EYA family members, such as Eya1 and Eya3, are required during mammalian organogenesis and through their phosphatase activity regulate genes encoding growth control, signaling and survival [20].

Overexpression of EYA2 has been shown to promote epithelial ovarian tumor growth [21] and breast cancer metastases [22]. On the other hand, enforced expression of Eya2 has been shown to trigger apoptosis in IL-3-dependant myeloid cells [23], and *EYA2* has been found to be aberrantly hypermethylated in most colorectal neoplasms [24], indicating the potential for *EYA2* promoter methylation as a marker of tumorigenesis. Against this background, we evaluated the expression of Eya2 in normal pancreas and in pancreatic cancer tissues and cell lines, examined the methylation and histone acetylation status of its promoter and determined the consequences of stably expressing *EYA2* in pancreatic cancer cells including effects on tumor growth and metastases in an orthotopic model and effects on gene expression.

## RESULTS

### Loss of EYA2 expression in pancreatic cancer

**Figure 1:**
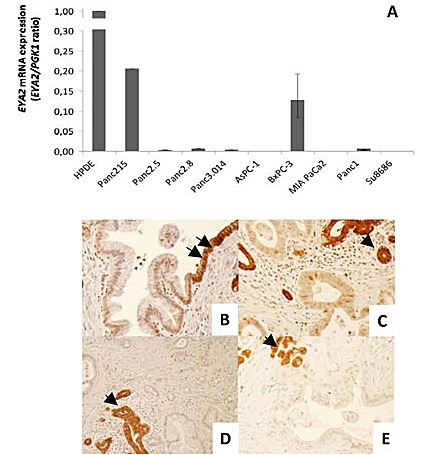
(A) EYA2 expression by real-time PCR in normal HPDE cells and nine pancreatic cancer cell lines (B-E) Representative figures of immunohistochemical staining of EYA2 with tissue microarrays. (B) Transition from normal ductal epithelium (arrows) to PanIN1. PanIN1 shows a decreased EYA2 expression. (C-E) Pancreatic ductal adenocarcinomas show a decrease or complete loss of EYA2 expression compared to entrapped normal ductal or acinar cells (arrows). Magnification: 10X.

Bioinformatic analysis of our Serial Analysis of Gene Expression data [3, 25] revealed *EYA2* mRNA as underexpressed in pancreatic cancers compared to pancreatic normal duct cells and HPDE, an immortalized non-neoplastic human pancreatic ductal epithelial line. Several hundred genes have been identified as silenced in pancreatic cancers by global gene expression analysis in prior studies [25, 26], but we focused on *EYA2* because of its putative functions and because it has not been recognized previously as underexpressed in pancreatic cancer. To confirm the SAGE data, we performed quantitative PCR analysis on HPDE and nine pancreatic cancer cell lines *i.e.* Panc215, Panc2.5, Panc2.8, Panc3.014, AsPC-1, BxPC-3, MIA PaCa2, Panc1 and Su8686. We found a 5-fold and a 7.8-fold decrease of *EYA2* expression in Panc215 and BxPC-3 cell lines compared to HPDE and very low (Panc2.8, Panc1) or virtually no expression in the seven other cell lines studied (Figure 1A). We then examined the expression of Eya2 protein in 189 primary pancreatic adenocarcinomas and adjacent normal and non-neoplastic pancreas by performing immunohistochemistry on tissue microarrays (Figure 1B-1E). Normal pancreas expression was localized to both the cytoplasm and nucleus but predominantly cytoplasmic (consistent with its phosphatase activity) with some cells displaying only cytoplasmic labeling. Complete loss of Eya2 protein expression was observed in the tumor cells of 63.5% of primary pancreatic adenocarcinomas (120 of 189 cases), while expression of Eya2 was found in normal ductal cells of 99.5% of cases. In addition to complete loss of expression, some pancreatic cancers retained only nuclear expression. We did not observe any pancreatic cancers with overexpression relative to normal pancreas. Patients with tumoral loss of Eya2 expression had significantly worse survival (median survival, 17.2 months) compared to patients whose cancers retained Eya2 expression (24.5 months, P=0.03), but Eya2 loss was not an independent predictor of survival when other factors associated with outcome (such as stage, differentiation, node status) were considered in a multivariate model (data not shown).

### EYA2 is epigenetically silenced in pancreatic cancer cell lines

**Figure 2:**
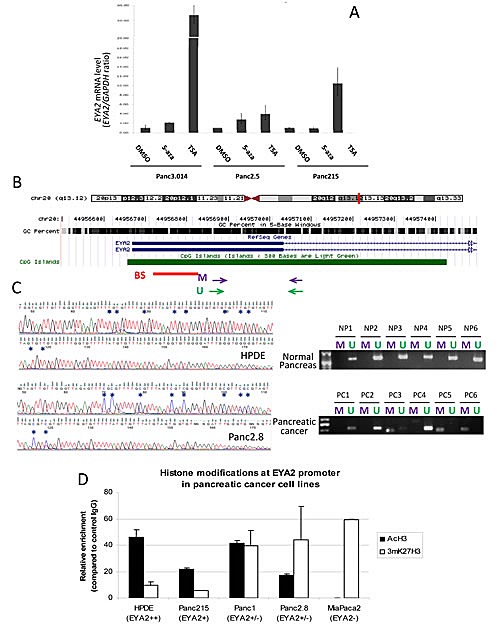
(A) Cells were treated with either 5-Aza-2'-deoxycytidine (5-Aza, 5 μM, 72hrs), trichostatin A (TSA, 0.3 μM, 24hrs) or DMSO prior to RNA extraction and analysis of EYA2 expression by real-time PCR. β-actin was used as an internal control for gene expression. (B) Chromosomal localization of EYA2 gene as represented by the UCSC Genome Browser. The GC percent in 5-base windows is shown for this region. The CpG island spanning the first exon (thick horizontal blue line) and the first part of the first intron (thin horizontal arrowed blue line) is represented in green. Horizontal red bar represents the region studied by bisulfite sequencing (BS). Purple and green arrows represent the primers used for MSP studies (M: Methylated, U: Unmethylated). (C) Representative bisulfite sequencing and MSP in normal pancreatic tissues (upper panels) and in primary pancreatic adenocarcinoma tissues (lower panels). (D) Chromatin immunoprecipitation was performed with specific antibodies against acetylated histone H3 (AcH3) and methylated K27 of histone H3 (mK27H3) in the Human Pancreatic Ductal Epithelial cell line HPDE and four pancreatic cancer cell lines harboring different levels of EYA2 expression (Panc215, Panc1, Panc2.8 and MiaPaca2). Enrichment of each histone modification at EYA2 promoter was assessed by qPCR and reported to the control (Normal rabbit IgG).

Since aberrant methylation is a common mechanism of gene silencing in pancreatic cancer [13], and *EYA2* has been shown to be hypermethylated in colon cancers [24], we investigated the role of epigenetic mechanisms in *EYA2* silencing. We first treated two *EYA2* non-expressing pancreatic cancer cell lines with the demethylating agent 5-aza-2'-deoxycytidine (5-azaC) and the histone deacetylase inhibitor, Trichostatin A (TSA). We found that 5-azaC increased *EYA2* mRNA expression in Panc3.014 and in Panc2.5, respectively by 2.1- and 2.8-fold, while TSA increased *EYA2* mRNA expression by 53.6 and 4.0-fold, respectively (Figure 2A). Treatment of the *EYA2*-expressing cell line Panc215 with 5-azaC had no significant effect on *EYA2* mRNA expression and treatment with TSA induced a 10.5-fold increase of *EYA2* mRNA expression (Figure 2A). We then examined *EYA2* promoter methylation by bisulfite sequencing in normal HPDE cells and five pancreatic cancer cell lines. We found the *EYA2* CpG island (Figure 2B) unmethylated in HPDE and the *EYA2*-expressing cancer cell line, Panc215, but it was methylated at most or all CpGs sequenced in the non-expressing Panc2.8 and A38-5 cell lines, respectively (Figure 2C), and remained unmethylated in the non-expressing Panc2.5 and Panc3.014 cells. We then used methylation-specific PCR (MSP) to assess *EYA2* promoter methylation in 53 pancreatic cancers (9 cell lines, 23 primary pancreatic cancer tissues and 21 xenografts of primary pancreatic cancers) and 58 normal pancreatic tissues. Complete DNA methylation of the *EYA2* promoter region amplified by MSP was identified in Panc2.8, Panc215, Panc2.5, and Panc3.014 cell lines, partial methylation in AsPC1 and only unmethylated templates in *EYA2*-expressing BxPC3 cancer cells. We found no evidence of promoter methylation in the non-expressing MiaPaca2, Panc1 and Su8686 cell lines suggesting that promoter methylation was not always required for loss of EYA2 expression. Overall, methylation of the *EYA2* promoter was found in 22.7% of pancreatic adenocarcinomas versus 6.9% of normal pancreatic tissues (p=0.019) (Figure 2C). Since the histone deacetylation inhibitor TSA could induce *EYA2* expression without 5-aza-dC in some cell lines, we further examined histone acetylation for its role in regulating *EYA2* expression (Figure 2A). Analysis of histone marks in pancreatic cancer cell lines by chromatin immunoprecipitation indicated a correlation between *EYA2* silencing and histone H3 deacetylation of the *EYA2* promoter (Figure 2D) where non-expressing cells showed a significant enrichment of the inactivating histone mark, 3mK27H3, while high-expressing cells showed a significant enrichment in acetylation of histone H3.

### EYA2 overexpression decreases pancreatic cancer cell proliferation in vitro

**Figure 3:**
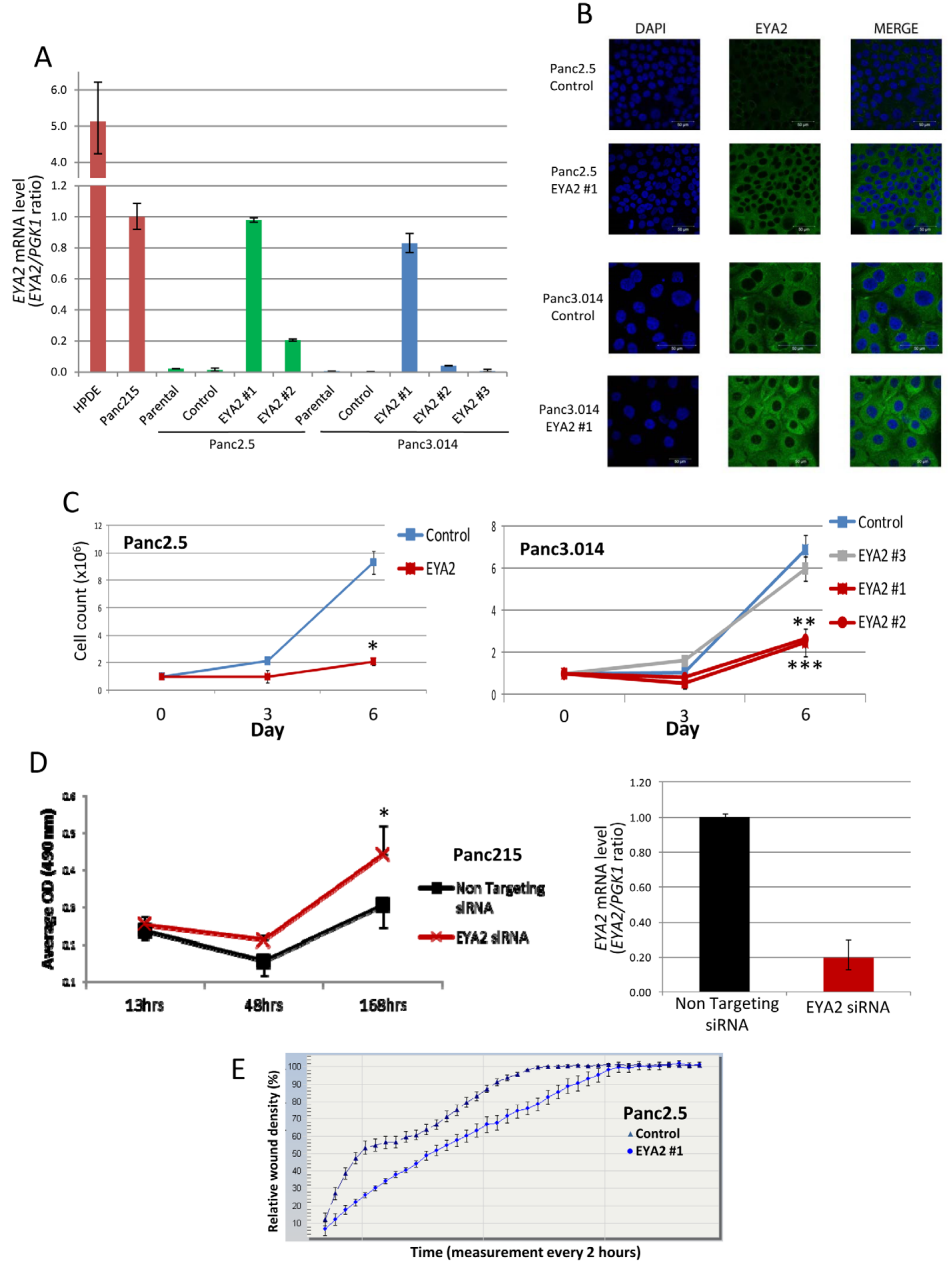
(A) Bar graph indicating the level of EYA2 mRNA expression in positive controls (normal HPDE cells and Panc215 cancer cells), non-transfected cancer cell lines (Panc2.5 and Panc3.014, parental), cells transfected with an empty vector (Panc2.5 and Panc3.014, control) and cells transfected with an EYA2 expressing vector (Panc2.5 EYA2 #1 and #2 and Panc3.014 #1, #2 and #3). (B) Confocal microscopy showing EYA2 localization and expression in Panc2.5 and Panc3.014 EYA4 expressing and control clones. (C) Proliferation curves of Panc2.5 and Panc3.014 control cells (blue curves) compared to EYA2 expressing stable transfectants (red curves) and EYA2 non-expressing stable transfectant (grey curve). 1×10^5^ cells were plated in 24-well plates and viable cells were then counted after three and six days. (D) Panc215 cancer cells were transfected with either a non-targeting siRNA or a siRNA targeting EYA2 24hrs after seeding. Cell proliferation was assessed by measuring absorbance at 490 nm after incubation of the cells with Cell Titer 96® Aqueous One Solution Reagent (Promega). *, P < 0.05; **; P < 0.01; ***. P < 0.0001. EYA2 specific knockdown was assessed by qRT-PCR. (E) Migration assay. Wound density was assessed every two hours for 72h using the Incucyte Live-Cell Imaging System

To investigate the function of EYA2 in pancreatic cells, we established stable *EYA2*-overexpressing clones from Panc2.5 and Panc3.014 pancreatic cancer lines using the pcDNA6.2/cLumio-DEST containing the *EYA2* transcript. Stable transfectants showed a significant overexpression of *EYA2* transcripts (Figure 3A). For subsequent experiments, we selected stable clones in which *EYA2* expression was comparable to the naturally expressing cancer cell line Panc215. Using immunocytofluorescence, we found that EYA2 protein was also overexpressed in these stable transfectants and showed perinuclear localization in the cells (Figure 3B).

We then compared cell proliferation in the stable transfectants expressing *EYA2*, to control transfected cells (empty pcDNA6.2/cLumio-DEST). Our results showed that *EYA2*-expressing Panc2.5 cells proliferated 4.4 times slower than control Panc2.5 cells (Figure 3C). Similarly, Panc3.014 *EYA2*-expressing clones #1 and #2 showed a 2.78 and 2.61-fold decrease in cell growth compared to control cells, respectively (Figure 3C). Panc3.014 stable clone #3, which expressed the same level of *EYA2* as Panc3.014 control cells, showed a similar proliferation rate compared to Panc3.014 control cells (Figure 3C). Conversely, we used a specific siRNA to inhibit *EYA2* expression in Panc215 cancer cells which yielded a 5.1-fold decrease of *EYA2* expression (Figure 3D) associated with a significant increase in cell proliferation (P=0.0015, Figure 3D).

We also compared cell migration in the stable transfectants expressing EYA2, to control transfected cells. Our results showed that *EYA2*-expressing Panc2.5 cells migrated 1.3 times slower than control Panc2.5 cells (Figure 3E).

### EYA2 overexpression prevents cell growth and metastasis in vivo

**Figure 4:**
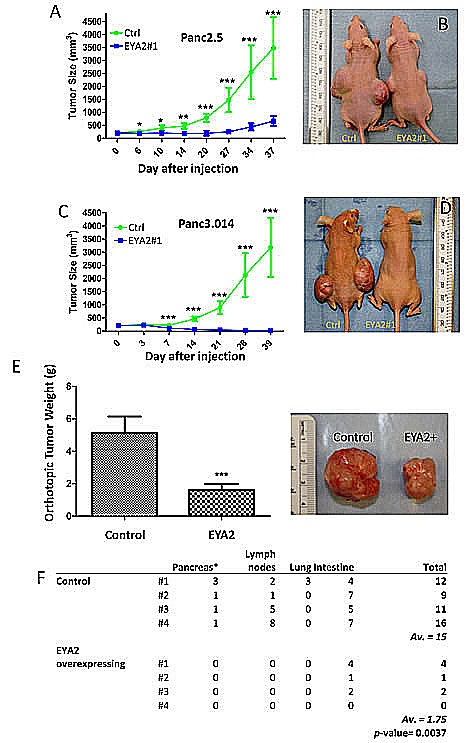
(A-D) Panc2.5 and Panc3.014 control and EYA2-overexpressing cells were used to perform subcutaneous xenografts in Nude mice. For each stable clone, five mice were xenografted and data represent Mean ± SD. *, P < 0.05; **; P < 0.01; ***. P < 0.0001. Tumor size is significantly smaller in mice xenografted with EYA2-overexpressing cells than in control cells. (E-F) Tumours obtained from subcutaneous xenografts of Panc2.5 control and EYA2-overexpressing cells were used to perform orthotopic xenografts in eight additional Nude mice. (E) Tumour weight 77 days after orthotopic implantation. (F) Total number of macroscopically observed metastases in mice xenografted with EYA2-overexpressing cells compared to control cells. The primary tumour in the pancreas was excluded from counting and does not appear in the table.

We further analyzed the role of EYA2 on tumor growth *in vivo* using an immunodeficient mouse model. Thirty-seven days after subcutaneous cell injection EYA2-overexpressing Panc2.5 cells showed a significant decrease in tumor size (7-fold, P<0.0001, Figure 4A and 4B) compared to Panc2.5 control cells. Similarly, EYA2-overexpressing Panc3.014 cells totally lost their potential to form a tumor *in vivo* (Figure 4C and 4D). To further evaluate the effects of EYA2 on tumor growth and on metastases, we implanted 1 mm^3^ subcutaneous tumors from Panc2.5 stable transfectant and control cells into the pancreas as orthotopic xenografts in the same immunodeficient mouse model. After 11 weeks, we found that EYA2-overexpressing Panc2.5 cells showed a significant decrease in tumor weight (3-fold, P<0.0001, Figure 4E) compared to control Panc2.5 cells. Additionally, we checked macroscopically for the presence of visible metastasis in lymph nodes, liver, lung and intestine as well as evidence of tumor cells in the pancreas, distant from the site of orthotopic grafting. We were not able to observe metastasis in the lungs, liver and lymph nodes of mice xenografted with EYA2-overexpressing tumors, while we observed several metastatic tumors in each mouse xenografted with control Panc2.5 tumors (Mean ± SD, 5.5 ± 4.4 tumors, P = 0.025).

### EYA2 overexpression in pancreatic cancer cells modulates gene expression

**Figure 5:**
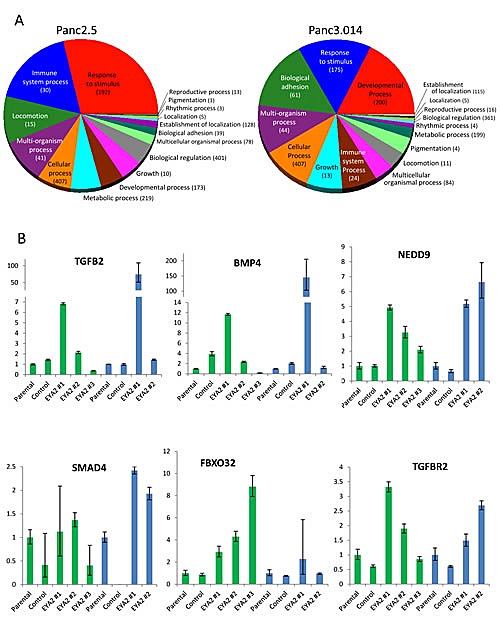
(A) Summary of differentially expressed genes in Panc2.5 and Panc3.014 EYA2-overexpressing clones compared to control clones. Statistical analysis of gene expression array data as well as Gene Ontology classification were performed with Partek Genomic Suite 6.4 software. (B) Quantitative RT-PCR showing the expression profile of genes from the TGFbeta pathway in EYA2-overexpressing clones compared to control clones and untransfected cells (Parental) in Panc2.5 (green bars) and Panc3.014 (blue bars) cells. Bands were quantified by densitometry.

To further understand the role of EYA2 on tumor growth and metastasis, we compared the transcriptomes of EYA2-overexpressing vs. control Panc2.5 and Panc3.014 cells (the same clones that were xenografted) using Affymetrix ST1.0 Exon Arrays. We identified one hundred and sixty genes differentially expressed by >2-fold in both of the EYA2-expressing cells relative to their corresponding control cells (stable empty vector-expressing Panc2.5 and Panc3.014 cells) (Supplemental Table 1), with additional genes showing expression changes in one of the two cell lines (data not shown). One hundred eighteen of these 160 genes contained a putative binding site for the EYA2 transcription factor in their promoter (Supplemental Table 1). We attempted to perform Chip analysis to identify EYA2 binding sites but available commercial antibodies did not enrich for putative EYA2 targets tested (data not shown). This list of genes induced by stable EYA2 expression in these cells included those responsible for growth and/or developmental processes (such as *IGF2*, *PTGS2, DZIP1*, and *NOTCH3*), response to injury (*TFF1*), the stem cell marker (CD133 (*PROM1)*, and genes related to adhesion (such as *ANTXR1*, *COL12A1*, *ITGB7*, *LAMA4* and *NID1*) and genes in the TGF beta signaling pathway or its downstream targets, either in Panc2.5 (*TGFΒR2, FBXO32*) or Panc3.014 (*SMAD4, CEACAM5*) cells or in both lines (*TGFB2, BMP4, THBS1, EID2*, and *NEDD9*) [27, 28] (Figure 5A). We examined the expression of seven of these genes by quantitative RT-PCR in the cell clones that were subcutaneously xenografted in nude mice as well as in one additional EYA2-overexpressing clone for each cell line (Figure 5B and Supplemental Figure 1). Additionally, we demonstrated overexpression of TGFBR2 protein by western blot in both EYA2-overexpressing clones (Supplemental Figure 2). We also observed a modest decrease in TGFΒR2 receptor phosphorylation in the Panc2.5-EYA2-overexpressing cells compared to control cells suggesting that the phosphatase activity of EYA2 reduced the proportion of TGFΒR2 phosphorylation in EYA2-expressing cells (Supplemental Figure 2).

## DISCUSSION

Here, we demonstrate that the majority of pancreatic cancers lose expression of the developmental transcription factor and phosphatase, EYA2. This loss of expression is associated with poorer patient survival and under expression is evident early during pancreatic tumorigenesis, even at the PanIN-1 stage. We also find evidence for epigenetic regulation of *EYA2* in pancreatic cancers with both aberrant DNA methylation and chromatin marks in different pancreatic cancers. Furthermore, *EYA2* expression had a dramatic reduction in tumor growth *in vivo* both in subcutaneous and in orthotopic models, in two different pancreatic cancer cell lines and in multiple clones. Indeed, no tumor growth was observed when EYA2-expressing Panc3.014 cells were xenografted. Furthermore, orthotopically implanted EYA2-expressing pancreatic cancers had many fewer metastases, beyond what would be expected by the reduced size of the primary tumor. Knockdown of EYA2 in EYA2-expressing pancreatic cancer cells resulted in an increase in cell proliferation and stably-expressing EYA2 pancreatic cancer cells had reduced cell migration.

Eya2 plays an important role in numerous species in regulating cell death and differentiation and is expressed throughout development [29-31] and influences human developmental processes [32].

In contrast to the tumor suppressive functions of EYA2 in pancreatic cancers, Zhang and collaborators [21] found that EYA2 is up-regulated in epithelial ovarian cancer, promotes tumor growth and its overexpression is associated with short overall survival and Farabaugh et al found EYA2 had prometastatic functions in breast cancer [22]. These data suggest that EYA2 has organ specific functions, perhaps depending on its cellular localization, and underlines the complexity of its double functions as a phosphatase and as transcription coactivator during differentiation and cell signaling processes [18]. These findings suggest that EYA2 may function as an oncogene or tumor suppressor depending on the cell type, an observation that has been observed for several other genes such as NOTCH1 [33]. Furthermore, it has been shown previously that EYA-induced cell death signals override survival factors and has many features typical of apoptosis [23]. Furthermore, prior studies have also found that Eya mutants overproliferate [18].

Consistent with its role as a transcription factor, we identified many genes that were either upregulated or downregulated in the EYA2-expressing pancreatic cancer cells, many of which were involved in developmental processes, cell growth and adhesion. Further work is needed to determine if these expression changes represent direct transcriptional effects or indirect effects of inducing EYA2 expression. The effects of stably-expressing EYA2 in pancreatic cancer cells we observed on TGF beta pathway gene expression may be of particular interest since the TGF beta signaling pathway is disrupted in many pancreatic cancers and as evidenced by the inactivating mutations in DPC4 in ~55% of pancreatic cancers and the occasional genetic inactivation of other pathway components (TGFBR2, TGFBR1, BMPR2)[3, 5, 34, 35]. Interestingly, EYA2 has recently been reported to influence TGF beta signaling in breast cancer cells [22], but in contrast to our findings in pancreatic cancer, these authors found that EYA2 functioned as a promoter of growth and metastases and a potential therapeutic target for inhibition. Our results indicate that EYA2 not only influences transcription of TGF beta pathway members but also affects phosphorylation of TGFBR2suggesting a dual role of EYA2 in the pancreas.

In summary, we find that silencing of EYA2 in the majority of pancreatic cancers; that such silencing is associated with poor outcome with pancreatic cancer; that EYA2 can suppress tumor growth in orthotopic models of pancreatic cancer, and that EYA2 signaling alters the expression of many genes involved in growth and development.

## MATERIAL AND METHODS

### Cell lines and tissue samples

Human pancreatic cancer cell lines Panc2.5 (Pa21C), Panc2.8 and Panc3.014 (Pa28C) were cultured as previously described [3]. HPDE cells were generously provided by Dr Ming-Sound Tsao (University of Toronto, Ontario, Canada).

EYA2-overexpressing clones were established by stably transfecting the pcDNA6.2/cLumio-DEST vector containing the EYA2 transcript in Panc2.5 and Panc3.014 pancreatic cancer lines. The Gateway cloning system (Invitrogen) was used to clone the EYA2 transcript variant ENSG00000064655 in the pcDNA6.2/cLumio-DEST vector.

Discarded frozen normal and neoplastic tissues were obtained from patients who had undergone pancreatic resection for pancreatic adenocarcinoma or pancreatic neuroendocrine neoplasm at Johns Hopkins Hospital. In addition, tissue microarrays (TMAs) of formalin-fixed paraffin-embedded tissues were retrieved from 189 patients who underwent surgical resection at our institution. Specimens were collected and analyzed with the approval of the Johns Hopkins Committee for Clinical Investigation.

### Immunohistochemistry

Immunohistochemical labeling was performed using HRP EnVision^+^ System (DAKO Corp.) on TMA slides as previously described [36]. Deparaffinized slides were subjected to heat-induced epitope retrieval using a steamer and DAKO Target retrieval solution (pH 6.0–6.2; DAKO Corp.). Slides were incubated with goat polyclonal anti-human EYA2 (Santa Cruz, clone N16, catalog number is 15097) diluted to 1:200. A semi-quantitative analysis of immunostaining was performed, quantifying both the percentage and intensity of labelled ductal cells. The intensity of immunolabeling of individual cells was scored on a scale from 0 (no staining) to 4 (strongest intensity). The percentage of labelled cells at each intensity level was multiplied by the corresponding intensity value to obtain an immunolabeling score. For survival analysis, immunolabeling was categorized as present or absent.

### Confocal microscopy

Confocal microscopy was performed on Panc2.5- and Panc3.014-EYA2-expressing and control clones grown on Lab-Tek Chamber Slide permanox (Nunc) as described previously [37]. Cells were incubated overnight with EYA2-specific antibody. Slides were mounted in mounting medium containing DAPI and visualized with a Zeiss LSM 710 confocal microscope (Carl Zeiss Microscopy); images were captured and analysed with the Zeiss Efficient Navigation software (ZEN, Carl Zeiss Microscopy). Cell fluorescence was measured with Image J software as previously described [38].

### Treatment with 5-aza-2'-deoxycytidine (5-aza-dC) and Trichostatin A (TSA)

Cells were treated with 5-aza-dC (Sigma Chemical Co) at 1μmol/L for 4 days and/or 1μmol/L of TSA for 24 hours as previously described [39].

### Quantitative reverse-transcriptase PCR (qRT-PCR)

Total RNA from cell lines was extracted using mirVana miRNA Isolation Kit (Ambion, Austin, TX) following the manufacturer's protocol and treated with DNA-free kit (Ambion). One μg of total RNA were reverse transcribed using Superscript^®^ III Reverse Transcriptase and random hexamers (Invitrogen Life Technologies; Carlsbad, CA) for qRT-PCR. *EYA2* cDNAs were quantified using SYBR Green PCR Master Mix for SYBR green I (Applied Biosystems, Foster City, CA). PCR was performed on an ABI7300 real-time thermocycler. Primers for *EYA2* were Fwd: 5'-GGACAATGAGATTGAGCGTGT-3' and Rev: 5'-ATGTCCCCGTGAGTAAGGAGT-3'. The housekeeping gene *ACTB* was used as a reference.

### Bisulfite modified sequencing and Methylation specific PCR

The methylation status of *EYA2* promoter was determined by bisulfite modified sequencing (BMS) and methylation-specific PCR (MSP) as previously described [40]. For BMS, primers were Fwd 5'-AGGAGGTTGGGTTTTGGTT-3' and Rev 5'-ATA AACAACTCCCCCC-3'. For MSP, DNA treated with SssI methylase (New England Biochemicals) and whole-genome amplified DNA (REPLI-g Mini Kit, Qiagen) were used as controls for methylated and unmethylated DNA, respectively. Primers were U: Fwd 5'-GGGAGGAGAAGGGGTTGGTTTTTTTG-3' and Rev 5'-CCTAAAATAAACACCACTAACAATA CTCACCA-3'; M: Fwd 5'-TTTCGGCGTAGGTAGTAGTCGC-3' and Rev 5'-GACCTAAAATAAACGCCGCTAACGA-3'.

### Chromatin Immunoprecipitation (ChIP)

ChIP was performed as previously described [41]. Briefly, cells were treated with 1% (v/v) formaldehyde and cross-links were quenched with glycine. Cells were rinsed with ice-cold PBS with protease inhibitors (Roche Applied Science), scraped and collected by centrifugation, before being resuspended in lysis buffer plus protease inhibitors. Chromatin was sheared with the Bioruptor system (Diagenode) to generate DNA fragments with an average size of ~200-400 bp. Antibodies to the repressive mark H3K27m3 and the active mark, acetylated H3, or normal IgG as a control were used. DNA was PCR amplified with primers targeting *EYA2* promoter (Fwd: CCTGCGCCTCTTTCTGGCACT and Rev: TCTGCCCTTGTGCCTTCCTGG).

### Small Interfering RNA Transfection

A siRNA targeting *EYA2* (ON-TARGETplus *SMART*pool EYA2, L-017233) and a non-targeting control siRNA were obtained from Dharmacon RNAi Technologies (Lafayette, CO). 1×10^5^ cells/well of Panc215 cells, were seeded in a 24 well plate and incubated for 24 hrs. Cells were transfected with *EYA2* siRNA or control siRNA (100 nmol/L) using DharmaFECT4 transfection reagent, incubated either for 48 hr or 7 days before cell counting. Specific *EYA2* knockdown was assessed by qRT-PCR as described above.

### Proliferation and migration Assays

For proliferation assays, 4,000 cells/well were seeded onto 96-well plates and incubated overnight. Cell proliferation was quantified either by adding 20 μl of CellTiter AQ_ueous_ One Solution (Promega) into each well containing 100 μl culture medium, and incubated for 2h at 37 °C before absorbance measurement at 490 nm or by counting the cells in each well with an automated cell counter (Invitrogen). Proliferation assays were done in sextuplicate in three separate experiments.

Cell migration was assessed by performing wound healing assays coupled with measurement of wound density every two hours for 72h with the Incucyte Live-Cell Imaging System (Essen Bioscience). Migration assays were done in sextuplicate in two separate experiments.

### Generation of pancreatic cancer xenografts and assessment of tumor growth and metastases

All animal experiments and maintenance conformed to the guidelines of the Animal Care and Use Committee of Johns Hopkins University and of the American Association of Laboratory Animal Care. Panc2.5 and Panc3.014 stable transfectants (overexpressing EYA2) and control (empty pcDNA6.2/cLumio-DEST vector) cells [10×10^6^ cells in 200 μL of 1/1 (v/v) PBS/Matrigel] were injected subcutaneously into male CD1 nu/nu athymic mice (Charles River, Wilmington, MA). One week after the tumor cell injection, subcutaneous tumor volumes (V) were measured weekly with digital calipers (Fisher Scientific) and calculated using the formula *V* = 1/2(*ab*^2^), where *a* is the biggest and *b* is the smallest orthogonal tumor diameter. After 3 weeks, the subcutaneous tumors from Panc2.5 stable transfectants and controls were harvested and cut into cubes of approximately 1 mm^3^ and orthotopic xenografts by surgical implantation were performed as described previously [42].

After 11 weeks, the mice were euthanized. The primary tumors were harvested. Each primary tumor was weighed with an analytical balance (Mettler Toledo, Switzerland). The tumor volume were measured with calipers of three orthogonal diameters (a, b and c) and calculated using the formula *V* = 1/2(abc). Spleen, pancreas, liver, intestine, colon, lymph node, peritoneum and lungs were inspected for grossly visible metastases and preserved in 10% formalin solution (Sigma-Aldrich) for histology. Regional lymph nodes were harvested and a histologic section examined if lymph node metastases were suspected from macroscopic inspection.

### Affymetrix Exon Arrays

Total RNA from Panc2.5 and Panc3.014 cells was extracted using mirVana miRNA Isolation Kit (Ambion, Austin, TX) per manufacturer's protocol before DNase treatment (Ambion).

The Affymetrix Exon Array ST1.0 (Affymetrix, Santa Clara, CA) was used to analyze gene expression profiles as previously described [25][43]. We are in compliance with the Minimum Information about a Microarray Experiment (MIAME) guidelines and have submitted our microarray data set to the NCBI's Gene Expression Omnibus under Accession Number GSE33282.

The presence of transcription factor binding sites within the promoters (from 2000 bp upstream the transcription start site) of potential target genes was assessed using the MAPPER Search Engine [44]. A customized model was built by supplying a multiple sequence alignment of binding sites for EYA2 transcription factor according to the previously described consensus binding sequence GTAANYNGANAYS (N = any nucleotide, Y = T/C, and S = C/G)[18, 45].

### Immunoprecipitation and western blotting

Total cellular extracts from Panc2.5- and Panc3.014-EYA2-expressing and control clones (200 or 100μg, respectively) were prepared using standard procedures[46] and immunoprecipitated overnight at 4°C with either 1μg of an anti-TGFbR2 antibody or normal Rabbit IgG, as a negative control, and EZ view Red protein A Affinity Gel (Sigma), according to the manufacturer's instructions. Western blotting was performed as described previously [46]. The membrane was probed with an anti-phospho-TGFbR2 or anti-TGFbR2 antibody. Relative quantification was performed using the Gel Analyst software.

### Statistics

Statistical analysis of gene expression array data was completed with Partek Genomic Suite 6.4 software. Raw Affymetrix intensity measurements of all probe sets were background corrected and normalized by the Robust Multichip Average method. Gene expression intensities were summarized by the one-step Tukey's biweight method. Survival rates were calculated by the Kaplan-Meier method, and statistical significance was examined by the log-rank test and the Cox proportional hazards regression model. One-way ANOVA analysis was performed to identify significant expression changes between EYA2-overexpressing and control pancreatic cancer cells and between control pancreatic cancer cells and the parental, non-transfected cell line. P-values of less than 0.05 were considered statistically significant. Statistical analyses were performed using SPSS version 11 (SPSS Inc., Chicago, Illinois).

## SUPPLEMENTARY FIGURES AND TABLE




