# Supplementary material for: Epigenetic silencing of EYA2 in pancreatic adenocarcinomas promotes tumor growth
Source: Oncotarget. 2014 Mar 22;5(9):2575–87. doi: 10.18632/oncotarget.1842 (PMC4058028; doi:10.18632/oncotarget.1842)
Supplement: Supplementary file 1 [file oncotarget-05-2575-s001.pdf]

Epigenetic silencing of *EYA2* in pancreatic adenocarcinomas promotes tumor growth – Vincent et al

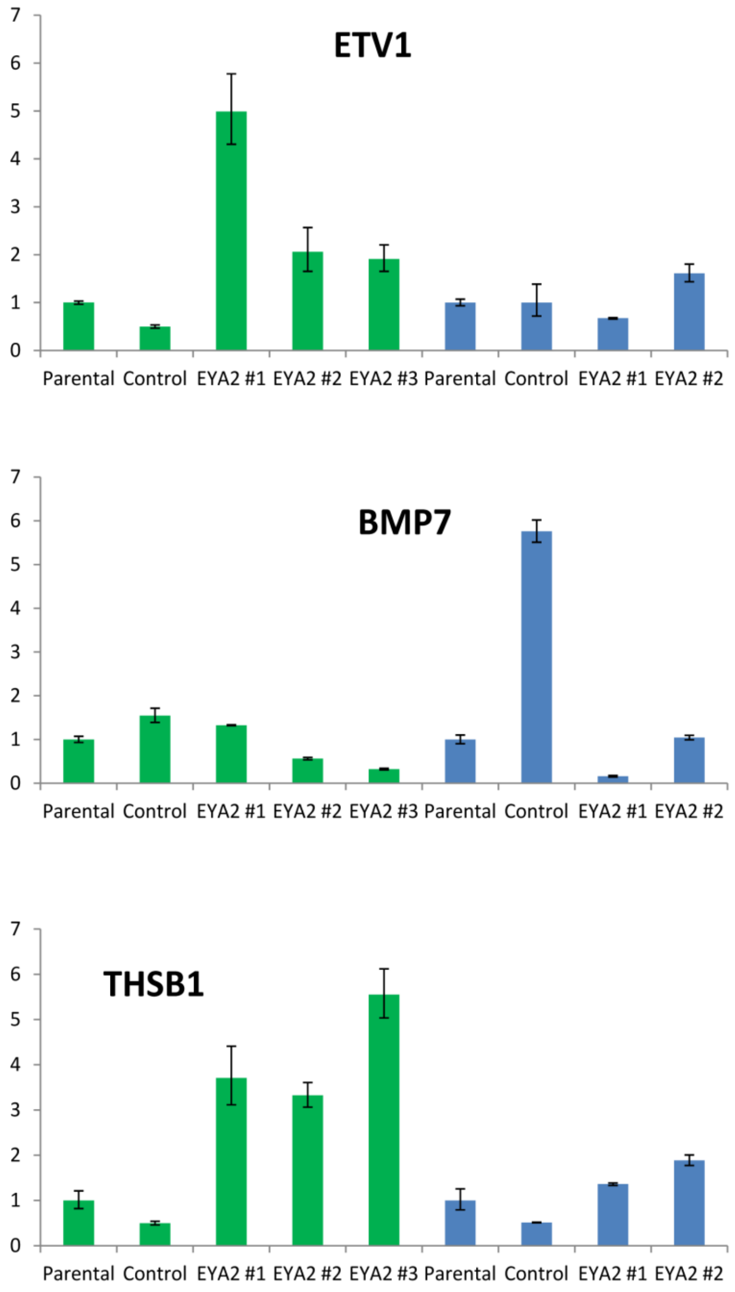

Supplemental Figure 1. Quantitative RT-PCR showing the expression profile of ETV1, BMP7 and THSB1 in EYA2-overexpressing clones compared to control clones and untransfected cells (Parental) in Panc2.5 (green bars) and Panc3.014 (blue bars) cells.

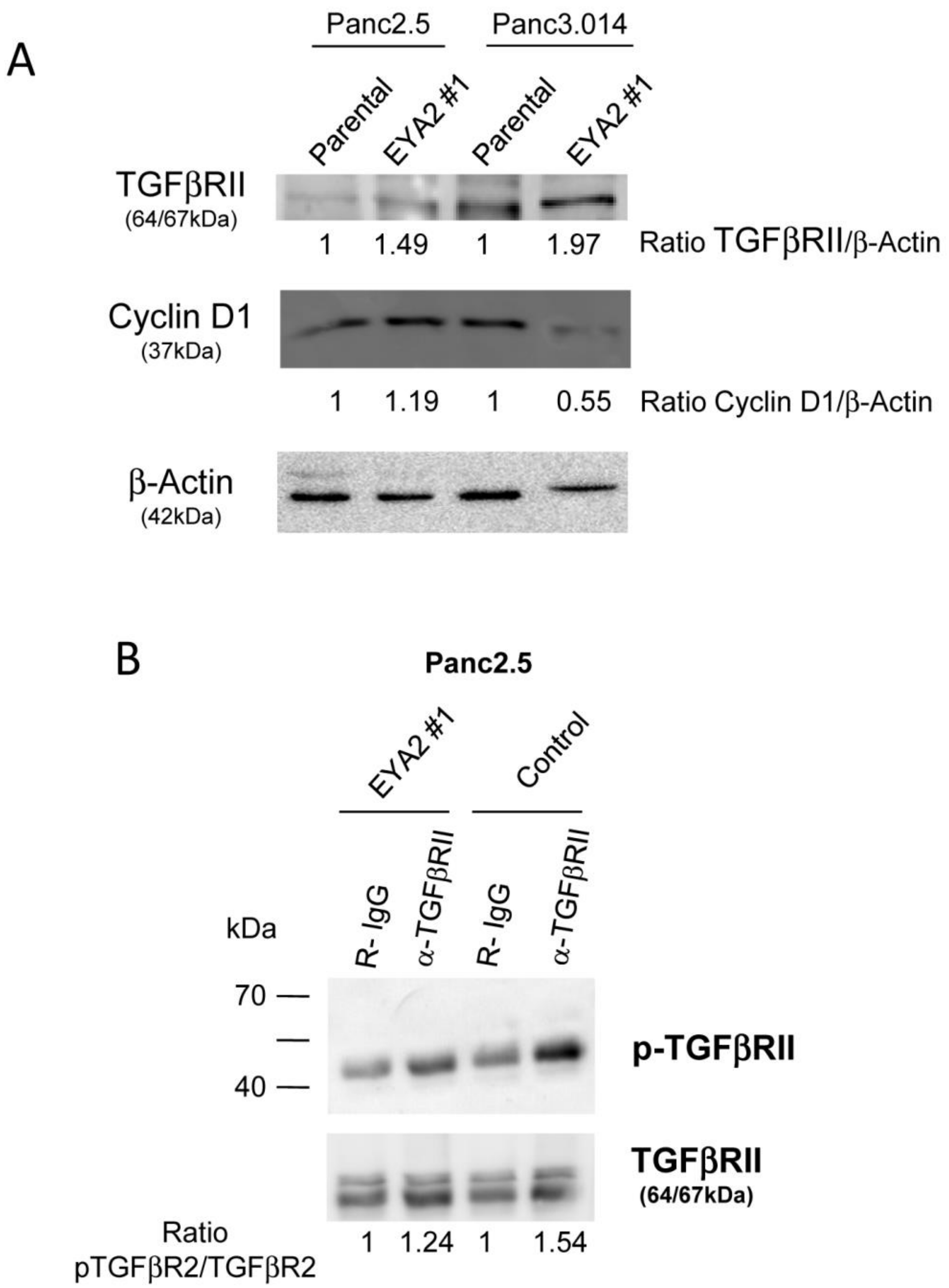

Supplemental Figure 2. (A) TGFBR2, Cyclin D1 and b-actin expression by western blotting. Bands were quantified by densitometry. TGFBRII/b-Actin and Cyclin D1/b-Actin ratios are shown under each blot where parental expression was arbitrarily set to 1. (B) TGFBR2 immunoprecipitation of total protein extracts was performed before western blotting using either an antibody anti-TGFbR2 or anti-pTGFbR2. Bands were quantified by densitometry. pTGFBR2/TGFBRII ratio are shown under each blot where immunoprecipitation with control IgG was arbitrarily set to 1.
